# Elevation of the Plasma Levels of TNF Receptor 2 in Association with Those of CD25, OX40, and IL-10 and HTLV-1 Proviral Load in Acute Adult T-Cell Leukemia

**DOI:** 10.3390/v14040751

**Published:** 2022-04-03

**Authors:** Megumi Kato, Naoki Imaizumi, Reiko Tanaka, Mariko Mizuguchi, Masaki Hayashi, Takashi Miyagi, Junnosuke Uchihara, Kazuiku Ohshiro, Junpei Todoroki, Kennosuke Karube, Hiroaki Masuzaki, Yuetsu Tanaka, Takuya Fukushima

**Affiliations:** 1 Laboratory of Hematoimmunology, Graduate School of Health Sciences, University of the Ryukyus, Nishihara 903-0215, Japan; k208872@eve.u-ryukyu.ac.jp (M.K.); reiko_tanaka@s5.dion.ne.jp (R.T.); 2 Laboratory of Molecular Genetics, Graduate School of Health Sciences, University of the Ryukyus, Nishihara 903-0215, Japan; imaizumi@med.u-ryukyu.ac.jp; 3 Department of Investigative Medicine, Graduate School of Medicine, University of the Ryukyus, Nishihara 903-0215, Japan; mizu@med.u-ryukyu.ac.jp; 4 Department of Hematology, Nakagami Hospital, Okinawa 904-2142, Japan; mhayashi@nakagami.or.jp; 5 Department of Hematology, Heart Life Hospital, Nakagusuku 901-2492, Japan; t.miyagi@heartlife.or.jp; 6 Department of Hematology, Naha City Hospital, Naha 902-8511, Japan; juchi@mte.biglobe.ne.jp; 7 Department of Hematology, Okinawa Prefectural Nambu Medical Center and Children’s Medical Center, Naha 901-1193, Japan; kazuoo2@at.au-hikari.ne.jp; 8 Department of Hematology, Chubu Tokushukai Hospital, Nakagami 901-2305, Japan; tookibunpei@yahoo.co.jp; 9 Department of Pathology and Laboratory Medicine, Graduate School of Medicine, Nagoya University, Nagoya 466-8550, Japan; karube@med.nagoya-u.ac.jp; 10 Division of Endocrinology, Diabetes, and Metabolism, Hematology, Rheumatology, Second Department of Internal Medicine, Graduate School of Medicine, University of the Ryukyus, Nishihara 903-0215, Japan; hiroaki@med.u-ryukyu.ac.jp

**Keywords:** ATL, HTLV-1, TNFR2, OX40, CD25, IL-10, biomarker

## Abstract

Adult T-cell leukemia/lymphoma (ATL) cells express TNF receptor type-2 (TNFR2) on their surface and shed its soluble form (sTNFR2). We previously reported that sTNFR2 levels were highly elevated in the plasma of patients with acute ATL. To investigate whether its quantitation would be helpful for the diagnosis or prediction of the onset of acute ATL, we examined the plasma levels of sTNFR2 in a large number of specimens obtained from a cohort of ATL patients and asymptomatic human T-cell leukemia virus type 1 (HTLV-1) carriers (ACs) and compared them to those of other candidate ATL biomarkers (sCD25, sOX40, and IL-10) by enzyme-linked immunosorbent assays (ELISA) and HTLV-1 proviral loads. We observed that sTNFR2 levels were significantly elevated in acute ATL patients compared to ACs and patients with other types of ATL (chronic, smoldering, and lymphoma). Importantly, sTNFR2 levels were significantly correlated with those of sCD25, sOX40, and IL-10, as well as proviral loads. Thus, the present study confirmed that an increase in plasma sTNFR2 levels is a biomarker for the diagnosis of acute ATL. Examination of plasma sTNFR2 alone or in combination with other ATL biomarkers may be helpful for the diagnosis of acute ATL.

## 1. Introduction

Adult T-cell leukemia/lymphoma (ATL) is a distinct peripheral T-cell malignancy associated with human T-cell leukemia virus type 1 (HTLV-1) infection [1,2,3,4]. The number of HTLV-1-infected individuals has been estimated to be more than 10 million worldwide [5]. The cumulative lifetime risk of developing ATL in asymptomatic HTLV-1 carriers (ACs) is estimated to be 3–5% [6]. The number of new ATL patients is approximately 1000 per year in Japan [7]. ATL is heterogenous and classified into four subtypes (acute, lymphoma, chronic, and smoldering) based on clinical manifestation and natural history [8]. The median survival time is 8–10 months in aggressive ATL, including acute, lymphoma, and unfavorable chronic ATL, and 2–4 years in indolent ATL, including favorable chronic and smoldering ATL [9,10].

Since no prophylactic vaccines or drugs against HTLV-1 infection and ATL are available at present, it has been suggested that the initiation of clinical treatment for patients from the early stage of ATL might be necessary to improve prognosis [11]. However, there are no methods to estimate the risk of developing ATL among ACs. One candidate approach is the evaluation of HTLV-1 proviral load (PVL) as HTLV-1-infected individuals with a PVL less than 4 copies per 100 peripheral blood mononuclear cells (PBMCs) do not tend to develop ATL [12,13]. Other putative biomarkers of ATL include elevated plasma levels of soluble interleukin (IL) 2 receptor (sCD25) [14], soluble OX40 (sOX40) [15], and IL-10 [16].

ATL cells express a wide variety of cell surface antigens associated with T-cell activation, such as CD25, OX40, CD30, MHC class-II, and TNF receptor type-2 (TNFR2). TNFR2 belongs to the TNFR superfamily, as does OX40 (CD134) [17], and functions as a signal molecule on the surface of a limited type of normal and tumor cells, including CD4^+^ regulatory T (Treg) cells and ATL cells [18,19]. The main function of TNFR2 is to support cell activation and survival upon ligation with its specific ligands, TNF-α and TNF-β [20,21]. sTNFR2 is released into the circulation after proteolysis of membrane TNFR2 [22]. Our recent proteome analysis of 1305 plasma proteins in samples from ACs and untreated ATL patients utilizing the SOMAscan method (SomaLogic, Inc., Boulder, CO, USA) identified some candidate ATL biomarkers including sTNFR2 [23].

Since our previous study examining the association between sTNFR2 levels and ATL was performed with a limited number of samples, in the present study, we tested a large number of samples from a cohort in Okinawa prefecture (Japan). Plasma sTNFR2 levels were analyzed in association with those of sCD25, sOX40, and IL-10, but also PVL. Here, we show that sTNFR2 is a definite biomarker of ATL, and discuss its potential use for the diagnosis and prediction of the progression to ATL from the AC stage.

## 2. Materials and Methods

### 2.1. Reagents

The culture medium used in this study was RPMI-1640 medium (Sigma-Aldrich, Inc., St. Louis, MO, USA) supplemented with 10% fetal bovine serum, 100 U/mL penicillin, and 0.1 mg/mL streptomycin. Recombinant human TNFR2 protein (rTNFR2) was purchased from Sino Biological, Inc., (Beijing, China). Goat anti-rat IgG labeled with FITC or HRP was purchased from BioLegend, Inc., (San Diego, CA, USA). A biotin-labeling kit was purchased from DOJINDO Molecular Technologies, Inc., (Kumamoto, Japan).

### 2.2. Blood Samples

Blood samples were obtained from ACs, ATL patients, and normal healthy volunteers living in Okinawa prefecture after obtaining informed consent. Clinical samples were procured from seven institutions in Okinawa prefecture (University of the Ryukyus Hospital, Heart Life Hospital, Nakagami Hospital, Naha City Hospital, Nanbu Medical Center, Chubu Tokushukai Hospital, and Kariyushi Hospital) from August 2014 to August 2021. ACs were confirmed with anti-HTLV-1 antibodies through the particle-agglutination method. ATL patients were diagnosed based on the criteria proposed by the Japan Clinical Oncology Group [8] and confirmed with a monoclonal integrated HTLV-1 proviral genome using Southern blot hybridization as described previously [24]. Plasma samples from 122 ACs and 136 ATL patients were collected. PBMCs and plasma samples were separated from heparinized blood samples by density-gradient centrifugation using Lymphocyte Separation Solution (Nacalai Tesque, Kyoto, Japan), and stored in gaseous nitrogen and at −80 °C, respectively, until used.

### 2.3. Generation of an In-House Human TNFR2 ELISA

For generation of in-house ELISA for quantitative determination of human TNFR2 in a large number of samples, we generated a library of anti-human TNFR2 mAbs from rats. Characterization of these mAbs in detail will be reported elsewhere (Kato et al. manuscript in preparation). In brief, mAbs specific for human TNFR2 were established from rats (WKAH/Hkm rats (Japan SLC, Inc., Shizuoka, Japan) immunized with either 10 µg/head rTNFR2 alone or together with cell lysates of a new human TNFR2-expressing syngeneic rat kidney cell line, WTR2, which was generated from a rat kidney epithelial cell line (W7KSV-1) [25] by transfection with a human TNFR2 expression plasmid generated by inserting human *TNFR2* cDNA into pEFneo [26] using the Neon Transfection System (Invitrogen Life Technologies, Carlsbad, CA, USA). Immune rat spleen cells were fused with SP2/0 myeloma cells by utilizing the HVJ Envelope Cell Fusion Kit (ISHIHARA SANGYO KAISHA, Ltd., Osaka, Japan), and selectively grown in an HAT medium. Antibody reactivity to human TNFR2 was screened using rTNFR2-coated ELISA plates along with secondary antibody goat anti-rat IgG-HRP. Hybridomas were cloned by limiting dilution, and each mAb was purified using protein-L or protein-G from culture supernatants. Some mAbs were biotinylated with NH_2_-Reactive Biotin (DOJINDO Molecular Technologies, Inc.). The IgG isotypes of the newly established 3–4 and 4–4 mAbs were IgG2a f as determined using a MonoAb-ID/SP Kit (Zymed Laboratories, Inc., San Francisco, CA, USA).

### 2.4. ELISA

Antigen quantitation of sOX40 and sCD25 by ELISAs was performed as described previously [15]. IL-10 was evaluated using a commercial kit for IL-10 (BioLegend). sTNFR2 was quantitated with a new ELISA established in the present study. As described in the text, to generate the in-house sTNFR2 ELISA, we selected a pair of mAbs from the newly established mAbs for performance comparable to that of the commercial human sTNFR2 ELISA Kit (R&D Systems, Inc., Minneapolis, MN, USA), which was utilized in the previous study [23]. To eliminate false-positive reactions related to human anti-xenogeneic antibodies, mouse IgG mAb anti-HIV-1 p24 (clone 2C2) [27] was added to the reaction buffer at a final concentration of 0.02 mg/mL. For detection of biotinylated detector antibodies, streptavidin-HRP conjugate (SDT GmbH, Baesweiler, Germany) was used. The substrate used was TMB solution (Sigma-Aldrich), while 2 N H2SO4 was used to terminate the reaction. Absorbance was read at 450 nm using a reference wavelength of 620 nm, with an automatic micro-plate reader (Bio-Rad, Hercules, CA, USA). All samples were analyzed in duplicate, and when the values of samples were out of the standard curves, the tests were repeated at an adequate dilution.

### 2.5. HTLV-1 PVL

PVL in PBMCs was measured with quantitative PCR using 100 ng genomic DNA (roughly equivalent to 1.0 × 10^4^ PBMCs) on a LightCycler 480 System II (Roche, Basel, Switzerland), as previously described [28]. HTLV-1 PVL was determined using the following formula: HTLV-1 tax copy number per 1.0 × 10^4^ PBMCs = ([tax copy number]/[β-actin copy number/2]) × 10^4^. All samples were analyzed in triplicate at the University of the Ryukyus Center for Research Advancement and Collaboration.

### 2.6. Statistical Analysis

Welch’s *t*-test was performed for statistical analysis using Prism8 software (GraphPad Software, San Diego, CA, USA). Results with *p*-values of less than 0.05 were considered significant. Correlations between the analyzed parameters were determined using Pearson’s correlation coefficient at *p* < 0.05. The cut-off values for analysis of sTNFR2, sCD25, and PVL were determined by receiver operating characteristic curves using JMP15 software (SAS Institute JMP Japan, Inc., Tokyo, Japan) [29].

### 2.7. Ethical Considerations

This study was approved by the Human Institutional Review Board and Institutional Animal Care and Use Committee on Clinical and Animal Research of the University of the Ryukyus (approval number: 1606). All blood samples and information were collected after obtaining written informed consent according to the Declaration of Helsinki.

## 3. Results

### 3.1. Establishment of a Quantitative Sandwich ELISA for Human sTNFR2

In order to cope with testing a large number of samples, we used our in-house ELISAs for sOX40 and sCD25 [15] and a newly established in-house ELISA for sTNFR2. The new sTNFR2 ELISA was generated using new mAbs, selected from six mAbs that recognized mutually different antigenic epitopes on human TNFR2 as determined by an antibody-binding competition assay (Table S1). Among them, a pair of mAbs (clones 3-4 and 4-4) was selected for the sandwich ELISA, because this combination had the best detection performance for sTNFR2 (Figure S1A). This ELISA was capable of specifically quantitating natural sTNFR2 in plasma with a similar performance to a widely used commercial TNFR2 ELISA kit (R&D Systems) that had been used for our previous study [23]. Using a commercial recombinant TNFR2 that had been calibrated with the TNFR2 ELISA kit (R&D Systems), the dynamic range of the new ELISA was shown to be 2–1000 pg/mL (Figure S1B). The performance of the new TNFR2 ELISA kit was not influenced by the presence of mouse anti-HIV-1 p24 IgG, at a concentration of 0.02 mg/mL which was used to neutralize human anti-mouse/rat natural antibodies (so-called HAMA) in plasma samples to prevent false-positive reactions in ELISA.

### 3.2. Quantitation of sTNFR2 Together with the Other Putative Biomarkers for Acute ATL

**Figure 1:**
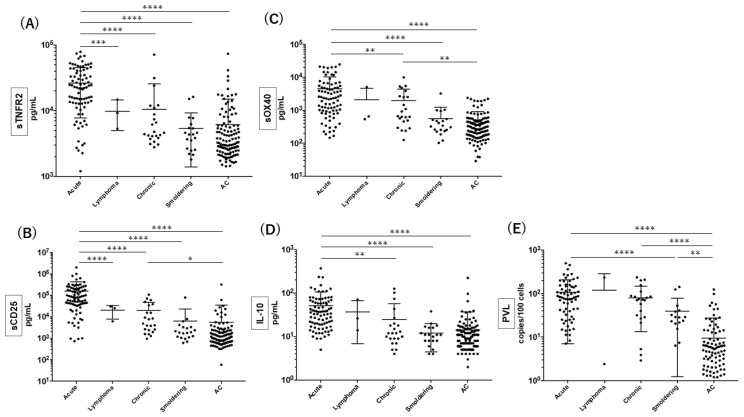
Comparison of plasma sTNFR2, sOX40, sCD25, and IL-10 levels and PVL among different types of ATL and ACs. Sample numbers of ATL and ACs tested were (**A**) 136 and 114, (**B**) 128 and 110, (**C**) 128 and 110, (**D**) 128 and 110, and (**E**) 108 and 113, respectively. **** *p* < 0.0001, *** *p* < 0.001, ** *p* < 0.01, and * *p* < 0.05.

According to our previous study [23] that suggested the potential clinical utility of quantifying plasma sTNFR2 levels for diagnosing acute ATL, we further validated the diagnostic value of sTNFR2 by comparison to other putative ATL biomarkers (sCD25, sOX40, and IL-10) in plasma and PVL. A large number of samples were collected from a cohort of ACs and ATL patients in Okinawa prefecture (Figure 1). In accordance with our previous study, plasma sTNFR2 levels were significantly higher in patients with acute ATL than in ACs and patients with other ATL types (Figure 1A). It was also clear that sTNFR2 levels were not significantly different between patients with chronic, smoldering, or lymphoma ATL and ACs. Similarly, plasma sCD25 levels were significantly higher in acute ATL patients than in ACs and patients with other ATL types (Figure 1B), although plasma sCD25 levels did not distinguish patients with smoldering and lymphoma ATL from ACs. With regard to sOX40, its levels were significantly higher in acute ATL patients than in ACs and smoldering ATL patients, but did not distinguish smoldering ATL patients from ACs (Figure 1C). In addition, plasma IL-10 levels were significantly higher in acute ATL patients than in ACs and chronic and smoldering ATL patients, but its levels were comparable between non-acute ATL patients and ACs (Figure 1D). Finally, PVL in PBMCs was highly elevated in patients with acute, chronic, and smoldering ATL compared to ACs, and PVL was comparable between acute and chronic ATL patients (Figure 1E).

These data demonstrated that plasma sTNFR2 levels are elevated in acute ATL, as observed for sCD25, sOX40, and IL-10 in plasma and PVL.

### 3.3. Close Relationship between sTNFR2, sCD25, sOX40, and IL-10 Levels and PVL

**Figure 2:**
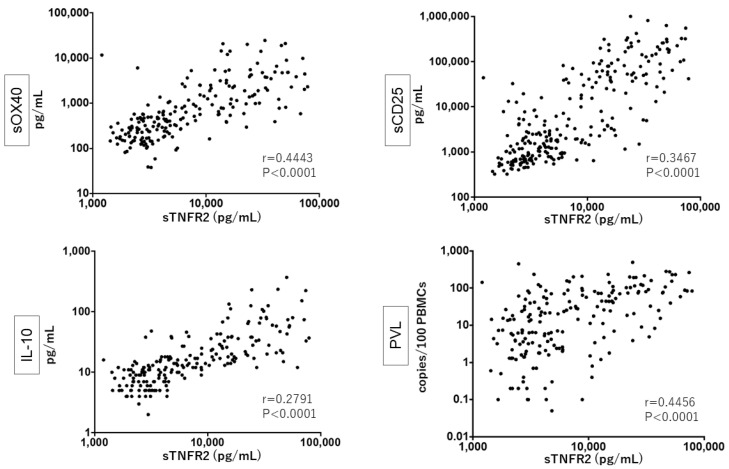
Correlation of plasma sTNFR2, sOX40, sCD25, and IL-10 levels and PVL in ATL patients and ACs. Samples tested were from 128 ATL patients and 110 ACs.

We examined whether there were correlations between the plasma levels of sTNFR2 with those of sOX40, sCD25, and IL-10 and with PVL in PBMCs among HTLV-1-infected donors. We observed clear, significant, and positive correlations (Figure 2). As speculated, all of these five factors were mutually and significantly correlated (Table S2). Altogether, our present data further supported the potential clinical utility of sTNFR2 for discriminating acute ATL patients from patients with other types of ATL and ACs, equivalent to sOX40, sCD25, IL-10, and PVL.

### 3.4. Grouping of the Status of HTLV-1-Infected Individuals

**Figure 3:**
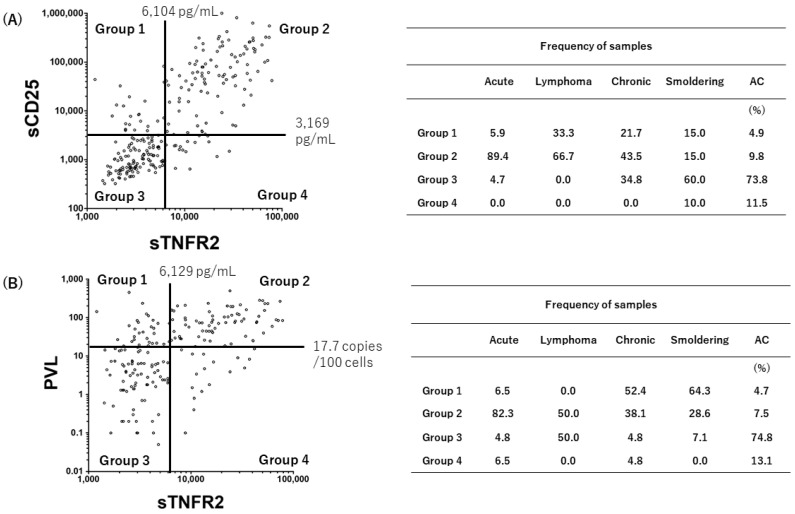
Grouping of ATL patients and ACs into four categories based on the scatter of the putative ATL biomarkers. Grouping data based on the sTNFR2/sCD25 scatter (**A**) and sTNFR2/PVL scatter (**B**) are shown on the left, and category frequencies (percentages) are summarized in the tables on the right. Each cut-off value was determined as described in the Section 2.

We further investigated the distribution of each ATL subtype and ACs on correlation diagrams drawn for either sTNFR2 vs. sCD25 or sTNFR2 vs. PVL (Figure 3). Putative quadrants were set according to the cut-off values of sTNFR2 (6104 pg/mL), sCD25 (3169 pg/mL), and PVL (17.7 copies/100 cells), which were calculated using receiver operating characteristic curves [29]. In the correlation diagram for sCD25 vs. sTNFR2 (Figure 3A), 89.4% of acute ATL patients were plotted in Group 2 (both high), which was in contrast to 73.8% of ACs in Group 3 (both low). On the other hand, in the correlation diagram for PVL vs. sTNFR2 (Figure 3B), 82.3% of acute ATL patients were distributed in Group 2 (both high), whereas 74.8% of ACs were in Group 3 (both low). In Figure 3B, there was a tendency that chronic and smoldering ATL patients were localized in Group 1 (high PVL, low sTNFR2).

These results suggested the present grouping method might be useful for the definition of ATL subtypes and subdivision of AC conditions.

## 4. Discussion

Using a large number of samples from a cohort of ACs and ATL patients in Okinawa, the present study confirmed that plasma sTNFR2 levels were highly elevated in patients with acute ATL compared to ACs and patients with other types of ATL. It is noteworthy that plasma sTNFR2 levels were significantly correlated with those of sCD25, sOX40, and IL-10, as well as with PVL. As far as we examined, the levels of these five factors were mutually correlated (Table S2). Therefore, it is now clear that the elevation of plasma sTNFR2 levels is a biomarker for acute ATL. The increased levels of those factors, especially in patients with acute ATL, may reflect tumor burden directly. On the other hand, the positive correlation between PVL with the plasma levels of sTNFR2, sCD25, sOX40, and IL-10 among HTLV-1-infected individuals may suggest that their levels are associated with the progression stages of HTLV-1 infection leading to ATL. In addition, from this point of view, it will be interesting to test patients with HTLV-1-associated myelopathy (HAM/TSP). Future studies, not only cross-sectional ones but also long-term observational studies, remain to be conducted.

The fact that plasma sTNFR2 levels are relatively higher than those of sCD25 or sOX40 in normal donors [15,23] suggests that the highly elevated sTNFR2 levels in acute ATL patients were produced not only from ATL cells but also from other cell types, including various immune cells [30], endothelial vascular cells [31], and brain cells [32]. Our in vitro study showed that normal monocytes and neutrophils release a considerable amount of sTNFR2 into the culture medium upon stimulation with the TLR4 ligand lipopolysaccharide and the inflammatory cytokine IL-8, respectively (Figure S2). Therefore, it is speculated that such innate immune cells produce substantial amounts of sTNFR2 in response to opportunistic infections, which generally occur in acute ATL patients [33,34]. In addition, since IL-10 augments the release of TNFR2 from monocytes [35,36], the elevated IL-10 levels in acute ATL patients may further stimulate sTNFR2 release in an autocrine or paracrine manner. Further studies are in progress to define the mechanisms underlying the elevation of plasma sTNFR2 levels in some ACs from the viewpoint of opportunistic infections and inflammation.

In the present study, we found a rare case of one donor who had been diagnosed with AC according to clinical subtype classification and who possessed high plasma sTNFR2 levels and PBMCs integrated with monoclonal HTLV-1 proviral DNA, suggesting that they could be defined as pre-ATL (Fukushima et al., manuscript in preparation). This observation provided further support for the hypothesis that elevated plasma TNFR2 levels are a possible biomarker for predicting the development of ATL in ACs. Further follow-up study of this and other cohorts is needed to demonstrate the prognostic value of sTNFR2 together with sOX40, sCD25, or IL-10 in plasma for aggressive ATL and for the prediction of high-risk ACs. In addition, the present study indicated that acute ATL patients above the cut-off level for sTNFR2 were clearly concentrated above the cut-off levels for sCD25 and PVL (89.4% and 82.3%, respectively) (Figure 3). In contrast, ACs under the cut-off level for sTNFR2 were clearly concentrated under the cut-off levels for sCD25 and PVL (73.8% and 74.3%, respectively). Again, these data supported the high clinical value of sTNFR2 as a biomarker for ATL.

TNFR2 has been considered a novel target for cancer immunotherapy and autoimmune diseases because it is aberrantly expressed by some tumor cells and normal cells including acute myeloid leukemia [37], Sezary syndrome [38], and Treg cells [39,40]. Since ATL cells express high surface levels of TNFR2 [23], it is speculated that TNFR2 can be a target of antibody drug therapy against ATL. Thus, our new anti-TNFR2 mAbs with either antagonistic or agonistic properties could be applied to the design of such an antibody drug. However, considerable care needs to be taken with this approach since TNFR2 is constitutively expressed on normal cells including monocytes, neutrophils, and Treg cells. Alternatively, sTNFR2 in the blood of acute ATL patients may interfere with exogenously administered anti-TNFR2 antibodies.

One question we need to address is whether membrane or soluble TNFR2 is involved in HTLV-1 infection or in the pathogenesis of ATL. It can be speculated that TNFR2 molecules on the surface of activated normal or HTLV-1-infected CD4^+^ T cells interact with HTLV-1 tax-induced TNF-β [41] and promote the survival of cells in autocrine and paracrine manners, or that sTNFR2 molecules with TNF-binding capacity may neutralize soluble forms of membrane-bound TNF-α or TNF-β to reduce TNFR1-mediated cell death [42]. Alternatively, as with Treg cells expressing TNFR2 for their expansion [43], HTLV-1-infected Treg cells may grow preferentially by virtue of TNFR2. Studies on these possibilities are currently in progress using our new mAbs including the antagonistic (1–11) and agonistic (4–7) clones (Kato et al., manuscript in preparation).

In conclusion, the present study showed that the abnormal elevation of plasma sTNFR2 is a definite biomarker for the diagnosis of acute ATL, suggesting that testing sTNFR2 alone or together with sCD25, sOX40, IL-10, and/or PVL will be helpful for the diagnosis of ATL. It is speculated that continuous screening of the plasma levels of sTNFR2 could be used to detect the early phase of acute ATL in patients with indolent ATL and ACs.

## Data Availability

Data is contained within the article or supplementary material.

## Supplementary Materials

The following supporting information can be downloaded at: https://www.mdpi.com/article/10.3390/v14040751/s1, Figure S1. Generation of a new quantitative ELISA for human sTNFR2; Figure S2. Production of sTNFR2 by PBMCs, monocytes, and neutrophils upon various stimuli; Table S1. Antibody binding competition assay; Table S2. Mutual correlation among sTNFR2, sOX40, sCD25, and IL-10 levels in plasma and PVL.
