# Supplementary material for: Elevation of the Plasma Levels of TNF Receptor 2 in Association with Those of CD25, OX40, and IL-10 and HTLV-1 Proviral Load in Acute Adult T-Cell Leukemia
Source: Viruses. 2022 Apr 3;14(4):751. doi: 10.3390/v14040751 (PMC9032861; doi:10.3390/v14040751)
Supplement: Supplementary file 1 [file viruses-14-00751-s001.zip › Figure S1.pdf]

(A) Comparison of sTNFR2 detection performance among different pairs of mAbs (OD_{450})

Capture mAbs	TNFR2 antigen	Detector 1-11	Detector 3-1	Detector 3-4	Detector 4-4	Detector 4-17
**1-11**	-	0.074	0.121	0.091	0.092	0.337
	+	0.082	0.134	0.510	0.823	0.659
**3-1**	-	0.061	0.084	0.065	0.098	0.153
	+	0.081	0.087	0.065	0.782	0.150
**3-4**	-	0.059	0.071	0.161	0.063	0.164
	+	0.568	0.068	0.161	<u>0.907</u>	0.785
**4-4**	-	0.059	0.083	0.069	0.064	0.157
	+	0.544	0.618	0.580	0.069	0.774
**4-17**	-	0.059	0.078	0.073	0.069	0.128
	+	0.468	0.085	0.522	0.859	0.161

(B) Standard curve drawn by the capture 3-4 and the detector 4-4 mAbs

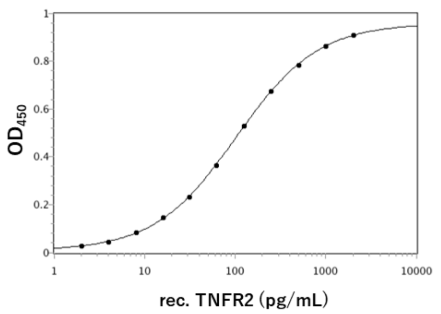

Figure S1. Generation of a new quantitative ELISA for human sTNFR2. (A) The sTNFR2 detection performance by sandwich ELISA was compered among different pairs of mAbs. The mean OD450 values in duplicate tests in the presence of no or 1,000 pg/mL rTNFR2 are shown. (B) sTNFR2 detection performance of the pair of mAbs clones 3-4 (capture) and 4-4 (detector) is shown as a standard curve of sTNFR2 titration at a range of 2~2,000 pg/mL.
