# Supplementary material for: Elevation of the Plasma Levels of TNF Receptor 2 in Association with Those of CD25, OX40, and IL-10 and HTLV-1 Proviral Load in Acute Adult T-Cell Leukemia
Source: Viruses. 2022 Apr 3;14(4):751. doi: 10.3390/v14040751 (PMC9032861; doi:10.3390/v14040751)
Supplement: Supplementary file 1 [file viruses-14-00751-s001.zip › Figure S2.pdf]

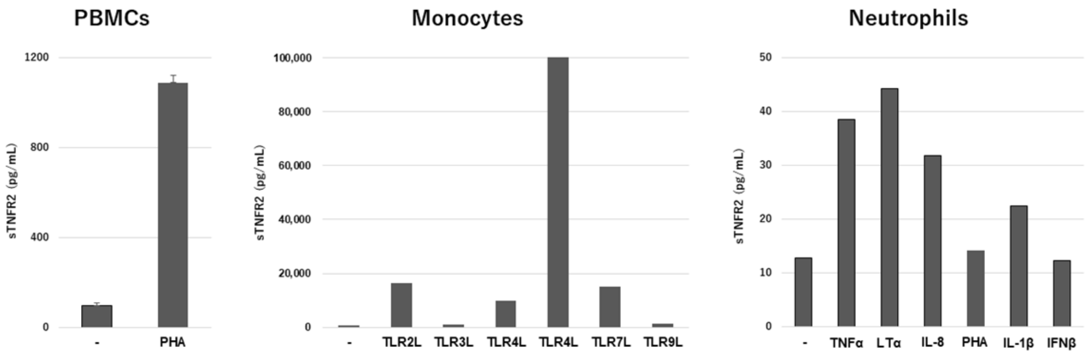

Figure S2. Production of sTNFR2 by PBMCs, monocytes and neutrophils upon various stimuli. PBMCs, purified monocytes and neutrophils from a normal donor were cultured in the presence or absence of phytohemagglutinin-P (PHA), various TLR ligands and cytokines for 24 hrs, and then the levels of sTNFR2 produced in the culture supernatants were determined by ELISA. The TLR ligands used were CL429 (for TLR2), PolyI:C (for TLR3), MPLA-SM (for TLR4), LPS (for TLR4), Gardiquimod (for TLR7) and ODN2395 (for TLR9).
