# Supplementary material for: Elevation of the Plasma Levels of TNF Receptor 2 in Association with Those of CD25, OX40, and IL-10 and HTLV-1 Proviral Load in Acute Adult T-Cell Leukemia
Source: Viruses. 2022 Apr 3;14(4):751. doi: 10.3390/v14040751 (PMC9032861; doi:10.3390/v14040751)
Supplement: Supplementary file 1 [file viruses-14-00751-s001.zip › Table S1.pdf]

Table S1. Antibody binding competiton assay

Blocker/Detector	1-11	3-1	3-4	4-4	4-7	4-17
**1-11**	+	-	-	-	±	-
**3-1**	-	+	±	-	+	±
**3-4**	-	-	+	-	+	-
**4-4**	-	-	-	+	±	-
**4-7**	-	-	+	-	+	-
**4-17**	-	-	-	-	+	+

The binding of each of biotinylated mAbs (0.1 μg/mL) to the rTNFR2 coated plates in the presence of unlabeled competitor mAbs (10 μg/mL) was determined by ELISA. +: strong inhibition, ±: intermediate inhibition, -: no inhibition.
