# Supplementary material for: Elevation of the Plasma Levels of TNF Receptor 2 in Association with Those of CD25, OX40, and IL-10 and HTLV-1 Proviral Load in Acute Adult T-Cell Leukemia
Source: Viruses. 2022 Apr 3;14(4):751. doi: 10.3390/v14040751 (PMC9032861; doi:10.3390/v14040751)
Supplement: Supplementary file 1 [file viruses-14-00751-s001.zip › Table S2.pdf]

Table S2. Mutual correlation among plasma levels of sTNFR2, sOX40, sCD25 and IL-10 and PVL in samples from ATL patients and ACs.

		sTNFR2	sOX40	sCD25	IL-10	PVL
sTNFR2	r		0.4443	0.3467	0.2791	0.4456
	P-value		<0.0001	<0.0001	<0.0001	<0.0001
sOX40	r	0.4443		0.3719	0.5101	0.3376
	P-value	<0.0001		<0.0001	<0.0001	<0.0001
sCD25	r	0.3467	0.3719		0.4964	0.5609
	P-value	<0.0001	<0.0001		<0.0001	<0.0001
IL-10	r	0.2791	0.5101	0.4964		0.2644
	P-value	<0.0001	<0.0001	<0.0001		0.0001
PVL	r	0.4456	0.3376	0.5609	0.2644	
	P-value	<0.0001	<0.0001	<0.0001	0.0001	
